# Suppression of chromosome instability by targeting a DNA helicase in budding yeast

**DOI:** 10.1091/mbc.E22-09-0395

**Published:** 2022-12-15

**Authors:** Molly R. Gordon, Jin Zhu, Gordon Sun, Rong Li

**Affiliations:** a Department of Cell Biology, Center for Cell Dynamics, Johns Hopkins University School of Medicine, Baltimore, MD 21205; b Mechanobiology Institute and; e Department of Biological Sciences, National University of Singapore, 117411; c Department of Biomedical Engineering and; d Department of Chemical and Biomolecular Engineering, Whiting School of Engineering, Johns Hopkins University, Baltimore, MD 21218; University of North Carolina, Chapel Hill

## Abstract

Chromosome instability (CIN) is an important driver of cancer initiation, progression, drug resistance, and aging. As such, genes whose inhibition suppresses CIN are potential therapeutic targets. We report here that deletion of an accessory DNA helicase, Rrm3, suppresses high CIN caused by a wide range of genetic or pharmacological perturbations in yeast. Although this helicase mutant has altered cell cycle dynamics, suppression of CIN by *rrm3∆* is independent of the DNA damage and spindle assembly checkpoints. Instead, the *rrm3∆* mutant may have increased kinetochore–microtubule error correction due to an altered localization of Aurora B kinase and associated phosphatase, PP2A-Rts1.

## INTRODUCTION

Heterogeneous aneuploid karyotypes are frequently detected within solid and hematopoietic tumors, suggesting that cancer cells have elevated chromosome segregation errors (Weaver and Cleveland, 2006). The increased propensity to missegregate chromosomes, referred to as chromosome instability (CIN), is a defining feature of cancer (Tijhuis *et al.*, 2019). CIN allows bursts of karyotypic diversity that enable tumor cells to sample chromosome constellations rapidly that endow the highest fitness advantage within the tumor microenvironment. As a result of this genome shuffling, increased intratumor heterogeneity in high-CIN tumors is correlated with poor patient prognosis and antineoplastic-drug resistance (Carter *et al.*, 2006; Choi *et al.*, 2009; Swanton *et al.*, 2009; Bakhoum *et al.*, 2011).

Paradoxically, data suggest that patients with the most complex and chromosomally unstable tumors have improved outcomes after chemotherapeutic treatment (Swanton *et al.*, 2009; Birkbak *et al.*, 2011; Roylance *et al.*, 2011; Zaki *et al.*, 2014; Jamal-Hanjani *et al.*, 2015). This finding suggests a “CIN threshold” beyond which the level of instability is incompatible with viability as cancer cells develop increasingly complex karyotypes (Thompson *et al.*, 2017). Given that it is a genetic driver of cancer progression and chemoresistance, CIN has been exploited as a target to halt cancer progression by synergistically increasing CIN to cause cancer cells to cross the viability threshold. An alternative approach would involve suppressing CIN to prevent the further evolution of a preexisting cancer or even possibly prevent the transformation of cancer cells by preventing karyotype diversity. However, there are currently no therapies based on suppressing CIN.

CIN can be caused by mutations that lead to increased mitotic errors. A prominent class of mutants causing CIN is those affecting microtubule–kinetochore attachments or pathways monitoring the attachment state between kinetochores and microtubules. Interestingly, the largest class of CIN mutants in yeast identified through genetic screens is those known to affect DNA replication and repair, and growing evidence suggests that even mild DNA replication stress (DRS) can lead to both structural and whole chromosome aneuploidy (Stirling *et al.*, 2011; Burrell *et al.*, 2013; Böhly *et al.*, 2019). DRS may even link early cancer-driving events such as oncogene mutation to a more pronounced CIN phenotype (Hills and Diffley, 2014; Gaillard *et al.*, 2015). It is unknown, however, whether there are common drug targets for suppressing CIN caused by various defects in cell cycle events.

Budding yeast may be an excellent model for identifying gene targets for suppressing CIN, as many of the genetic pathways involved in high-fidelity chromosome segregation are evolutionarily conserved (Yuen *et al.*, 2007; Stirling *et al.*, 2011). Using a single-cell quantitative yeast screening tool, we found that deletion of an accessory helicase, Rrm3, led to CIN suppression in severe CIN mutants within the DNA replication class of mutants. Remarkably, *rrm3∆* also suppresses CIN in mutants directly affecting mitotic processes. We further investigated how the loss of a single DNA helicase could buffer CIN under these diverse perturbations.

## RESULTS

### Loss of Rrm3, but not the other Pif1 helicase, leads to broad chromosome instability suppression

Our study was originally aimed at using a quantitative chromosome transmission fidelity (qCTF) assay to assign CIN rates to CIN mutants identified in previous genetic screens in yeast (Yuen *et al.*, 2007; Stirling *et al.*, 2011; Zhu *et al.*, 2015), with the goal of identifying nonmitotic pathways that affect CIN. The qCTF assay works by detecting the loss of an ectopic chromosomal fragment containing the left arm and centromeric sequence of chromosome III, resulting in the accumulation of GFP molecules (Zhu *et al.*, 2015). We screened nonessential gene knockouts (KOs) from the CIN mutant list, excluding those classified with the gene-ontology annotation of “Kinetochore, Spindle and Cell Cycle” (Stirling *et al.*, 2011). Our initial list contained 338 nonessential genes of which 324 were successfully introduced into the qCTF yeast strain (Supplemental Table S3). The 48 mutants with the highest CIN rates were further confirmed as to genotypes and validated with at least seven additional biological replicates in the qCTF assay (Supplemental Figure S1). Mutants of DNA replication and repair proteins constituted the largest group of the confirmed CIN mutants and gave rise to some of the highest CIN rates, further supporting the notion that DRS is a potent inducer of numerical aneuploidy (Gaillard *et al.*, 2015).

**FIGURE 1:**
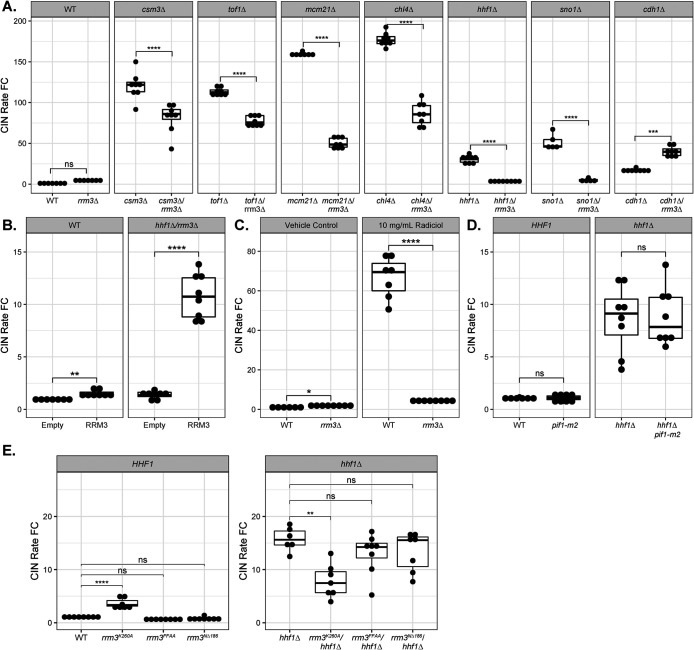
The effect of Rrm3 helicase mutation on multiple CIN mutants. The qCTF assay was used to measure CIN rates, which are expressed as a fold change (FC) relative to a WT strain for a selection of mutants in the presence or absence of *RRM3* (A), to test the effect of exogenous plasmid-based *RRM3* on the CIN rate of a *hhf1∆ rrm3∆* double mutant compared with an empty control plasmid (B), to test the effect of 10 µg/ml Radicicol or an equivalent volume of DMSO (vehicle control) on WT and *rrm3∆* cells (C), a *pif1-m2* mutation in the *hhf1∆* background (D), or to measure CIN rates of *rrm3^K260A^*, *rrm3^FFAA^*, or *rrm3^N∆186^* in a WT or *hhf1∆* background (E). Note that *y*-axis values differ for *hhf1∆* between experiments in panels A, D, and E, which is due to experimental variation. Lower and upper hinges on the box plot correspond to the first and third quartiles (the 25th and 75th percentiles). The median of the data is represented as a line between the upper and lower hinges. The upper whisker extends from the hinge to the largest value no further than 1.5 × IQR from the hinge (where IQR is the interquartile range or the distance between the first and third quartiles). The lower whisker extends from the hinge to the smallest value at most 1.5 × IQR of the hinge. Statistical analysis was performed via a Tukey post hoc test with the following significance cut-off: not significant (n.s.): *p* > 0.05, *: *p* ≤ 0.05, **: *p* ≤ 0.01, ***: *p* ≤ 0.001, ****: *p* ≤ 0.0001.

Two DRS mutants, *tof1∆* and *csm3∆*, led to an over 100-fold increase in the CIN rate, the two highest CIN rates measured after *ctf18∆*—deleting a gene encoding a key subunit of DNA replication factor C (Mayer *et al.*, 2001). Of note, the CIN rate in the *ctf18∆* mutant was highly variable between multiple screened colonies, which were also heterogeneous in size, indicative of the possible existence of phenotypic modifiers. Tof1 and Csm3 are present at the replisome as a dimer and contribute to programmed replication pausing at repetitive regions such as the centromere and the ribosomal DNA (rDNA) array (Bando *et al.*, 2009). Sister chromatid cohesion was shown to be perturbed in the absence of the Tof1/Csm3 pausing complex (Fernius and Marston, 2009), and this can be antagonized by Rrm3, an accessory 5′ to 3′ DNA helicase thought to act as a “molecular sweepase” that removes proteins tightly bound at programmed pause sites (Ivessa *et al.*, 2003; Mohanty *et al.*, 2006, 2009). *rrm3∆* leads to increased replisome pausing and a slower replication program, which is detectable through flow cytometry DNA content analysis of asynchronous cultures (Supplemental Figure S2). In line with previous findings, *rrm3∆* only moderately elevates the CIN rate over the wild-type (WT) control (Admire *et al.*, 2006). Interestingly, *rrm3∆* led to a 25.8% and 38% reduction in CIN rates in the *tof1∆* or *csm3∆* mutant background, respectively (Figure 1A).

To determine if the CIN suppression by *rrm3∆* was specific to the *tof1∆* and *csm3∆* mutants, we combined *rrm3∆* with several high-CIN mutants affecting diverse components of cell division pathways such as kinetochore formation, sister chromatid cohesion establishment, spindle assembly checkpoint (SAC), and the anaphase promoting complex (APC). Surprisingly, when *RRM3* was deleted in most (12 out of 20 screened) of these high-CIN backgrounds, such as *hhf1∆* (a histone H4 deletion mutant), CIN rates decreased compared with those single mutants (Figure 1A, Table 1). To confirm that CIN suppression by the *rrm3∆* mutant was due to loss of *RRM3*, we introduced centromeric plasmid-based *RRM3* with its promoter and terminator sequences into the *hhf1∆ rrm3∆* double mutant. The *RRM3* plasmid reversed CIN suppression in the double mutant (Figure 1B). *rrm3∆* also elevated the CIN rate in several mutants, most notably *cdh1∆*, gene deletion of an activator of the APC (Figure 1A). These findings suggest that, unexpectedly, deletion of *RRM3* is capable of suppressing CIN outside of its established role in regulating DRS.

**TABLE 1:** The effect of Rrm3 deletion on high CIN mutants.

Gene name	Single mutant CIN FC (i.e. *csm3∆*)	*rrm3∆* CIN FC (i.e., *csm3∆/rrm3∆*)	Percentage change	Significance
*CSM3*	120.4	89.3	–25.9	****
*TOF1*	113.4	77.2	–32	****
*SIC1*	38.7	24	–37.9	n.s.
*MAD2*	15.5	8.7	–44.3	n.s.
*MAD1*	10.4	5.2	–50	*
*CHL4*	177.3	86.4	–51.3	****
*HHF2*	8.2	3.4	–58.8	**
*MCM21*	159	50	–68.6	****
*HHF1*	30.2	3.7	–87.9	***
*BIM1*	10.9	1.1	–90	n.s.
*SNO1*	51.7	4.9	–90.6	***
*PSH1*	7.6	0.5	–93.1	****
*BUB1*	46.3	55.8	20.4	***
*DDC1*	1.9	2.3	24.2	n.s.
*SGO1*	29.7	61.8	107.6	****
*MPH1*	3.4	7.1	112.3	n.s.
*CDH1*	17.6	40	127.3	****
*RAD23*	2.8	8	182	**
*HHT2*	2.4	6.9	189.3	****
*HHT1*	1.6	8	418	****

*Notes:* CIN rates are expressed as a fold change (FC) relative to a WT qCTF strain. The percentage change between the single mutant CIN FC and double mutant CIN rate FC is shown, with negative values denoting a percentage decrease.

Furthermore, we tested whether *rrm3∆* suppresses CIN due to perturbations of functional complexes containing some of the proteins tested above. *sno1∆* causes CIN due to perturbations of the mRNA levels of the neighboring essential gene *CTF13* (Gordon *et al.*, 2021). The CIN rate in this mutant was decreased 90.6% by *rrm3∆* (Figure 1A). *CTF13* is a subunit of the CBF3 complex that binds the CDEIII sequence within the yeast centromere (Biggins, 2013). Treatment of yeast with the Hsp90 inhibitor Radicicol was previously shown to induce CIN by altering the stoichiometry of CBF3 components at the centromere (Chen *et al.*, 2012). *rrm3*∆ markedly reduced CIN in the presence of Radicicol (Figure 1C). Depletion of histone H4 is known to increase kinetochore declustering, alter centromere chromatin structure, and cause erroneous kinetochore–microtubule attachments (namely syntelic attachments) leading to activation of the SAC (Saunders *et al.*, 1990; Bouck and Bloom, 2007; Murillo-Pineda *et al.*, 2014). It is thought that this occurs specifically with depletion of histone H4 and not H3 because H4 binds to the centromeric H3 variant, Cse4, as a prerequisite for forming the centromeric nucleosome, which may be disrupted upon altered H4 stoichiometry (Bouck and Bloom, 2007). *rrm3∆* suppressed CIN caused by deletions of either histone H4 gene, *hhf1∆* or *hhf2∆*, by 87.9% or 58.8%, respectively (Supplemental Figure S3A). *rrm3∆* also significantly suppressed the CIN rate in a *psh1∆* mutant, deletion of a ubiquitin ligase gene involved in both the degradation of excess Cse4 molecules not bound to the centromere and the proper segregation of extrachromosomal plasmids (Supplemental Figure S3B; Hewawasam *et al.*, 2010; Ranjitkar *et al.*, 2010; Deyter *et al.*, 2017; Metzger *et al.*, 2017). On the other hand, *rrm3∆* increased CIN in mutants for either histone H3 gene, *hht1∆* or *hht2∆* (Supplemental Figure S3C). These results suggest that *rrm3∆* suppresses CIN associated with centromere or kinetochore defects.

Rrm3 and Pif1 constitute the Pif1 helicase family in budding yeast (Bessler *et al.*, 2001; Bochman *et al.*, 2010). Rrm3 and Pif1 have antagonistic roles at some genomic loci such as telomeres and rDNA repeats but may also act cooperatively at other loci such as centromeres (Muellner and Schmidt, 2020). We therefore tested if a *pif1* mutant would similarly lead to CIN suppression. The yeast Pif1 protein has a mitochondrial and nuclear isoform, and complete deletion of the *PIF1* ORF leads to an increased frequency of petite mutants lacking mtDNA (Lahaye *et al.*, 1991; Schulz and Zakian, 1994). To mitigate the effect on mtDNA and overall growth rate, we generated the *pif1-m2* mutant, in which the second methionine in the *PIF1* ORF was mutated, leading to the depletion of only the nuclear Pif1 isoform (Schulz and Zakian, 1994)*.* Unlike the *rrm3∆* mutant, *pif1-m2* does not lead to significant CIN suppression in an *hhf1∆* mutant background (Figure 1D), suggesting that CIN suppression is uniquely associated with the loss of Rrm3.

### Chromosome instability suppression requires inactivation of Rrm3 helicase activity but not checkpoint-mediated cell cycle delays

To gain insight into how deletion of Rrm3 leads to CIN suppression, we generated mutations within different domains of the Rrm3 protein to determine which was sufficient for CIN suppression. A mutation in lysine 260 (*rrm3^K260A^*) disrupts the ATPase activity and thus helicase function (Ivessa *et al.*, 2000). Rrm3 also contains a PCNA-interacting protein box (PIP-box) that allows Rrm3 to interact with the replication machinery. We disrupted the PIP-Box by mutating two conserved phenylalanines to alanines (*rrm3^FFAA^*), which was previously shown to prevent the physical interaction between Rrm3 and PCNA (Schmidt *et al.*, 2002). Since *rrm3∆* mutants are known to have persistent activation of the DNA Damage Response (DDR), we also generated a truncation of the first 186 amino acids of the Rrm3 N-terminal sequence (*rrm3^N∆186^*) as this truncation does not activate the DDR (Rossi *et al.*, 2015; Syed *et al.*, 2016). Each of the above *rrm3* mutants, expressed under the endogenous promoter and terminator sequence within the *RRM3* locus on chromosome VIII, was combined with the high-CIN mutant *hhf1∆*. qCTF analysis showed that only the *rrm3^K260A^* helicase-dead mutant suppressed CIN in the *hhf1∆* background, similarly to the complete *RRM3* deletion (Figure 1E). *rrm3^K260A^* also suppressed CIN in three other high CIN mutants, *sno1∆*, *chl4∆*, and *tof1∆* (Supplemental Figure S4). These results suggest that disruption of the Rrm3 helicase function is necessary and sufficient for CIN suppression, whereas disrupting PCNA-binding or preventing DDR activation was not.

**FIGURE 2:**
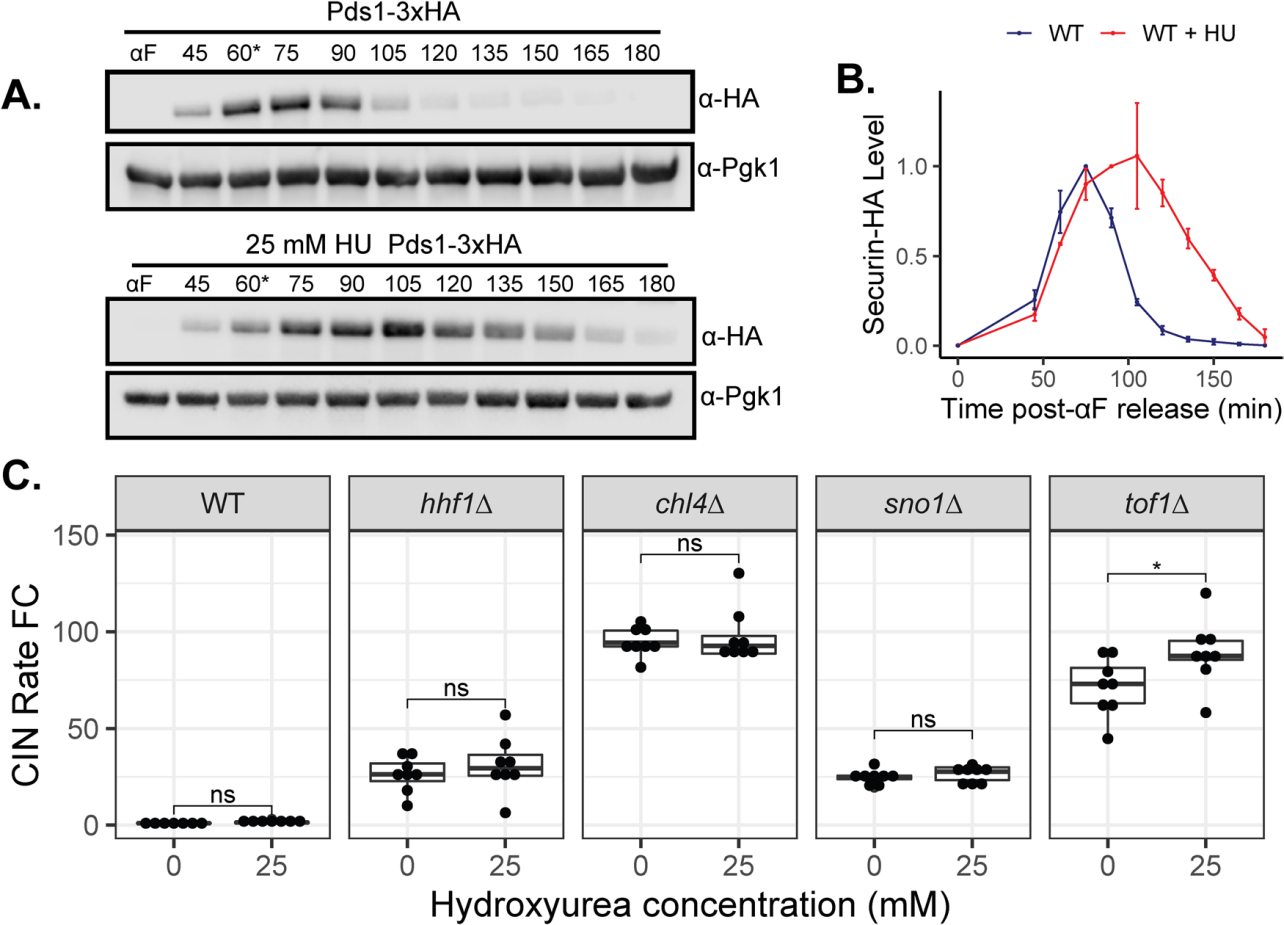
Pharmacologically induced S phase delay does not suppress CIN. Representative immunoblot (A) and quantification (B) of Pds1-3xHA in WT cells released from α-factor (αF) arrest into media supplemented with 25 mM hydroxyurea (HU). Asterisks mark when αF was added back to the medium to prevent cells from entering a subsequent cell cycle. WT securin degradation plot overlaid as reference (for more details see Figure 3C). Error bars represent the standard error of the mean (SEM). (C) qCTF CIN rate measurements of WT, *tof1∆*, *chl4∆*, *sno1∆*, and *hhf1∆* mutants cultured in 25 mM HU or an equivalent volume of vehicle control for 24 h. Box plots and statistical analysis are as in Figure 1.

Previous studies showed that delaying cell cycle progression in S or M phases could help alleviate mitotic errors associated with spindle defects (Vinton and Weinert, 2017). Both *rrm3∆* and *rrm3^K260A^* lead to an S phase delay as assessed by flow cytometry (Supplemental Figure S2). We thus investigated if a cell cycle delay, such as that observed in *rrm3∆*, is sufficient for CIN suppression. To delay S phase, we treated cells with the ribonucleotide reductase inhibitor hydroxyurea (HU) at 25 mM—a concentration sufficient to increase the fraction of cells in S phase and stabilize the anaphase inhibitor securin (Figure 2, A and B). This HU concentration was also previously shown to activate the DDR evidenced through Rad53 phosphorylation (Pike *et al.*, 2004). Importantly, 25 mM HU treatment was not sufficient to increase CIN under the WT condition (Figure 2C). This treatment, however, did not significantly reduce CIN caused by *chl4∆*, *hhf1∆*, or *sno1∆*, and even led to a subtle but significant increase in CIN rate in the *tof1∆* mutant background (Figure 2C). This finding suggests that slowing down DNA replication is insufficient to reproduce the CIN suppression achieved by *rrm3∆*.

**FIGURE 3:**
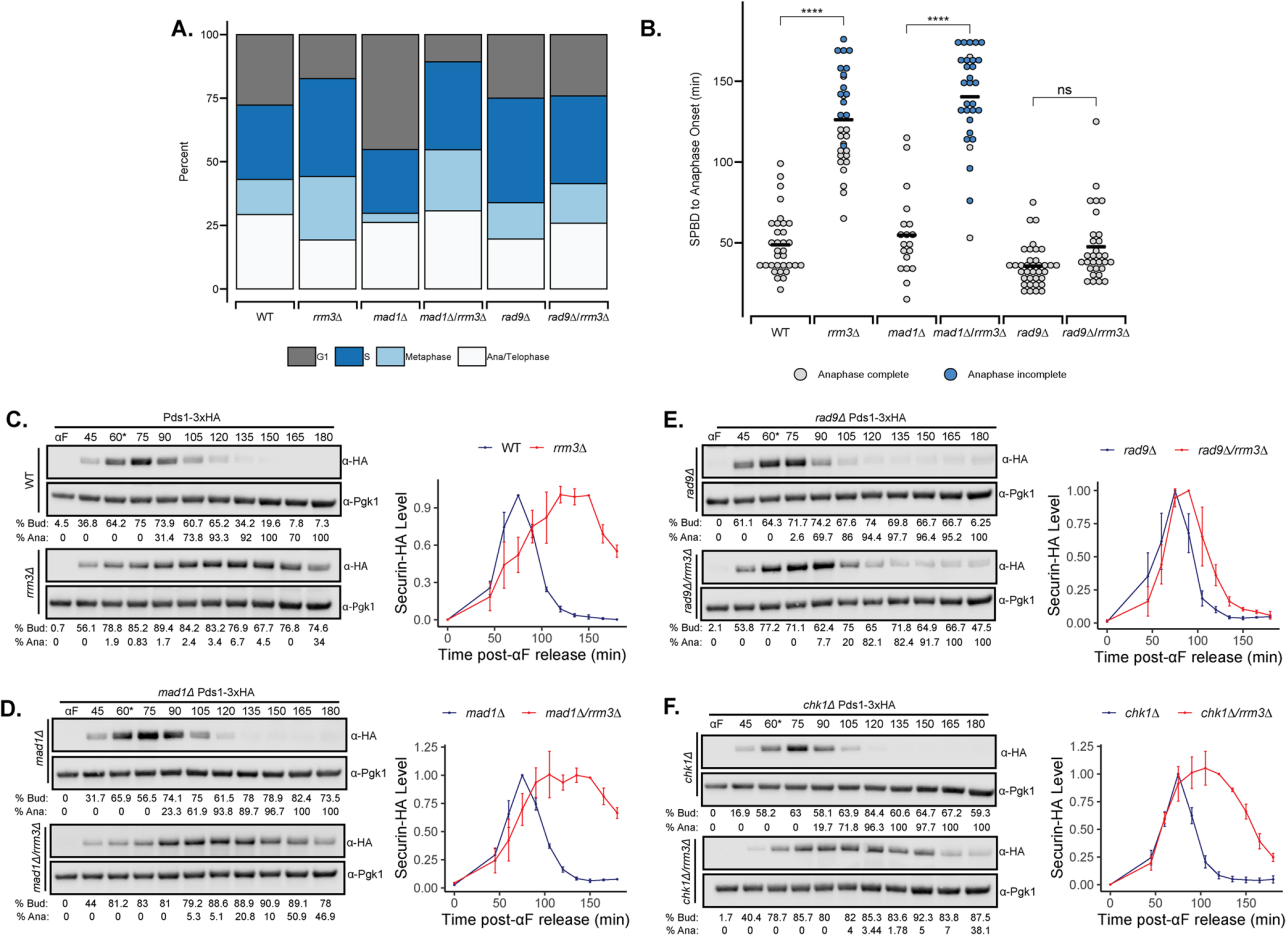
*rrm3∆* mutants exhibit a metaphase-to-anaphase delay corresponding to Rad9-dependent securin stabilization. (A) Quantification of asynchronous cell populations using Spc42-mCherry to categorize cell cycle stage in WT, *rrm3∆*, *mad1∆*, and *mad1∆/rrm3∆, rad9∆*, and *rad9∆/rrm3∆* mutants. *n* > 50 cells/condition. (B) Quantification of time between spindle pole body duplication (SPBD) and anaphase onset in the same strains as A. Black bar represents the mean of the data. *n* = 19–33 cells/condition. Blue data points denote cells that did not complete anaphase by the end of the movie. *p*-values for all plots were calculated from Tukey post hoc test. (C–F) Representative immunoblots and quantification for Pds1-3xHA and % budded cells and % cells undergoing anaphase in αF synchronized WT, C, *mad1∆*, D, *rad9∆*, E, and *chk1∆*, F, strains in the presence and absence of Rrm3. Asterisk indicates the time point at which αF was supplemented back into the medium.

To observe whether *rrm3∆* causes a delay in other phases of the cell cycle, we used a fluorescent spindle pole body (SPB) marker (Spc42-mCherry) to measure spindle distance as an indicator of cell cycle stage. An S phase delay in the *rrm3∆* mutant was evidenced by an increase in the fraction of cells with short bipolar spindles (<1.5 μm), not yet aligned with the mother-bud axis (Figure 3A). *rrm3∆* also increased the fraction of cells with a metaphase spindle, identified based on spindles not exceeding 3 μm in length while aligned with the mother-bud axis (Figure 3A). Since a metaphase delay associated with *rrm3∆* was not previously reported, we further examined mitotic progression by time-lapse microscopy of cells expressing GFP-tagged Ndc80, an outer kinetochore component, and Spc42-mCherry. Live imaging confirmed that *rrm3∆* cells had a delay in the metaphase-to-anaphase transition (Figure 3B). This delay was also associated with securin stabilization (Figure 3, C and D; Supplemental Figure S5, A and B), persistence of the nuclear cohesin pool (Supplemental Figure S5, C and D), and a delay in the release of the mitotic exit regulator Cdc14 phosphatase from the nucleolus (Supplemental Figure S5, E and F).

Cell cycle checkpoints, such as the SAC and DDR, converge on securin stabilization to prevent anaphase entry (Sanchez *et al.*, 1999; Agarwal *et al.*, 2003; Kim and Burke, 2008; Palou *et al.*, 2017; Pardo *et al.*, 2017). For example, the DDR kinase Chk1 directly phosphorylates securin to prevent APC-mediated degradation (Sanchez *et al.*, 1999). Both securin stabilization and the metaphase-to-anaphase delay associated with the *rrm3∆* mutant persisted in a *mad1∆* background defective in SAC function (Figure 3, A, B, and D). Previous studies have identified that, within the DDR, Rad9 is required for phosphorylation of Rad53 in the *rrm3∆* mutant, a modification required for checkpoint function (Ivessa *et al.*, 2003; Schmidt and Kolodner, 2004). Indeed, securin stabilization or metaphase-to-anaphase delay was not observed in the *rrm3∆ rad9∆* double mutant (Figure 3, A, B, and E); however, securin stabilization was unaffected in *chk1∆* strains (Figure 3F).

**FIGURE 4:**
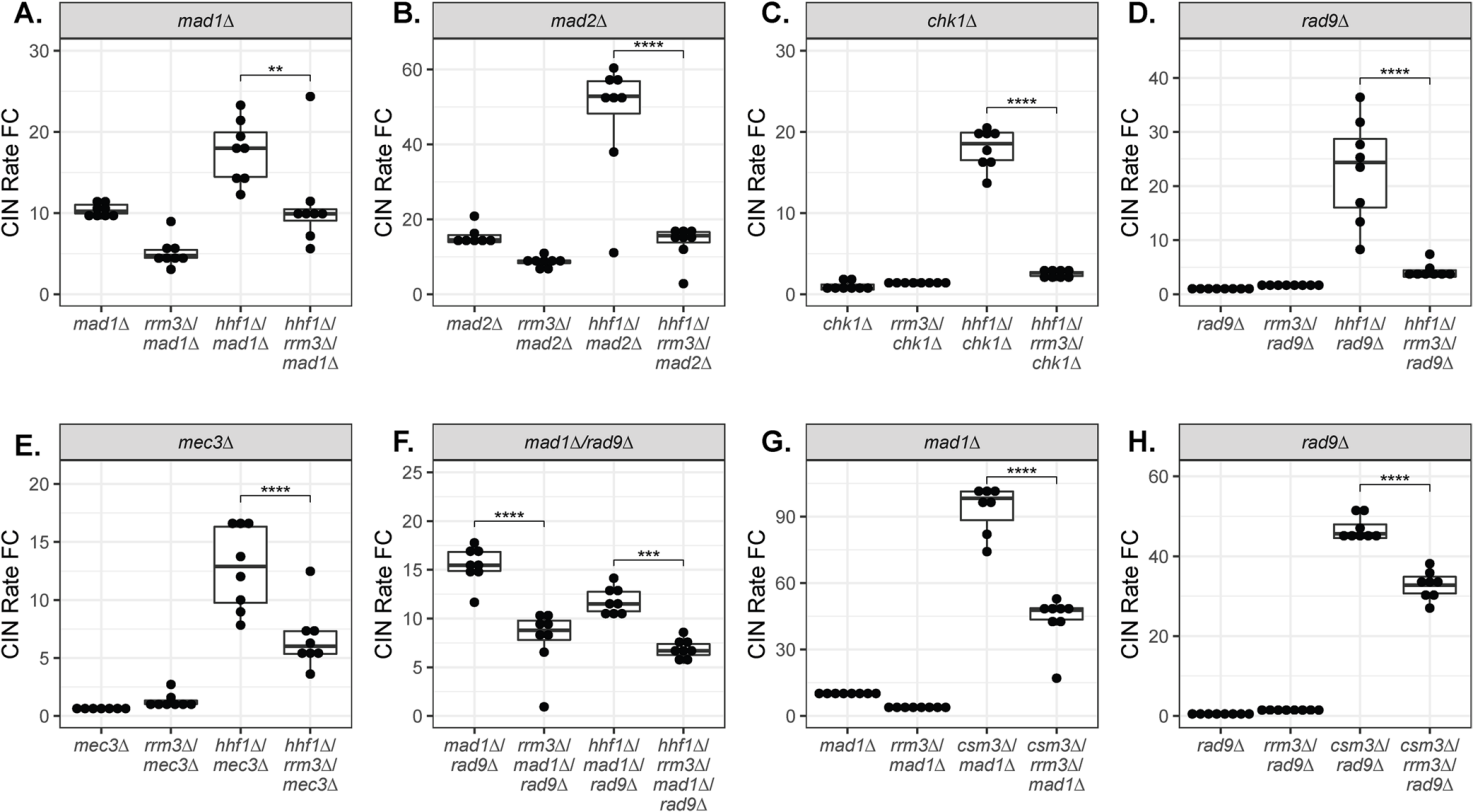
Neither the DDR nor the SAC is required for *rrm3∆*-dependent CIN suppression. qCTF measurements of the effect of *mad1∆* (A), *mad2∆* (B), *chk1∆* (C), *rad9∆* (D), *mec3∆* (E), or a *mad1∆/rad9∆* double deletion (F) on CIN rate in the *hhf1∆* mutant background and *mad1∆* (G) or *rad9∆* (H) on CIN rate in the *csm3∆* mutant background. Box plots and statistical analyses are as in Figure 1.

Next, we tested whether the mitotic delay generated by cell cycle checkpoints was necessary for *rrm3∆*-dependent CIN suppression. *rrm3∆* still suppressed *hhf1∆*-induced CIN in the absence of Chk1, Mad1, or Mad2, consistent with the observation that these proteins are not required for the cell cycle delay in *rrm3∆* (Figure 4, A–C). Surprisingly, *rad9∆* also did not prevent *rrm3∆*-dependent CIN suppression in the *hhf1∆* background despite being required for the metaphase-to-anaphase delay in *rrm3∆* cells (Figure 4D). The same result was also obtained with deletion of *MEC3*, encoding a DDR sensor within the 9-1-1 complex, which, like Rad9, is also necessary for Rad53 phosphorylation in the *rrm3∆* mutant (Figure 4E; Schmidt and Kolodner, 2006). We also tested simultaneous deletion of both *RAD9* and *MAD1* and found that abolishing both checkpoints together did not prevent CIN suppression by *rrm3∆* in the *hhf1∆* background (Figure 4F). We further confirmed that neither Rad9 nor Mad1 was required for *rrm3∆*-dependent CIN suppression in a second high CIN mutant, *csm3∆* (Figure 4, G and H). These results suggest that CIN suppression by *rrm3∆* depends neither on the DDR nor the SAC checkpoint.

### *rrm3∆* enhances Aurora B-mediated error correction

**FIGURE 5:**
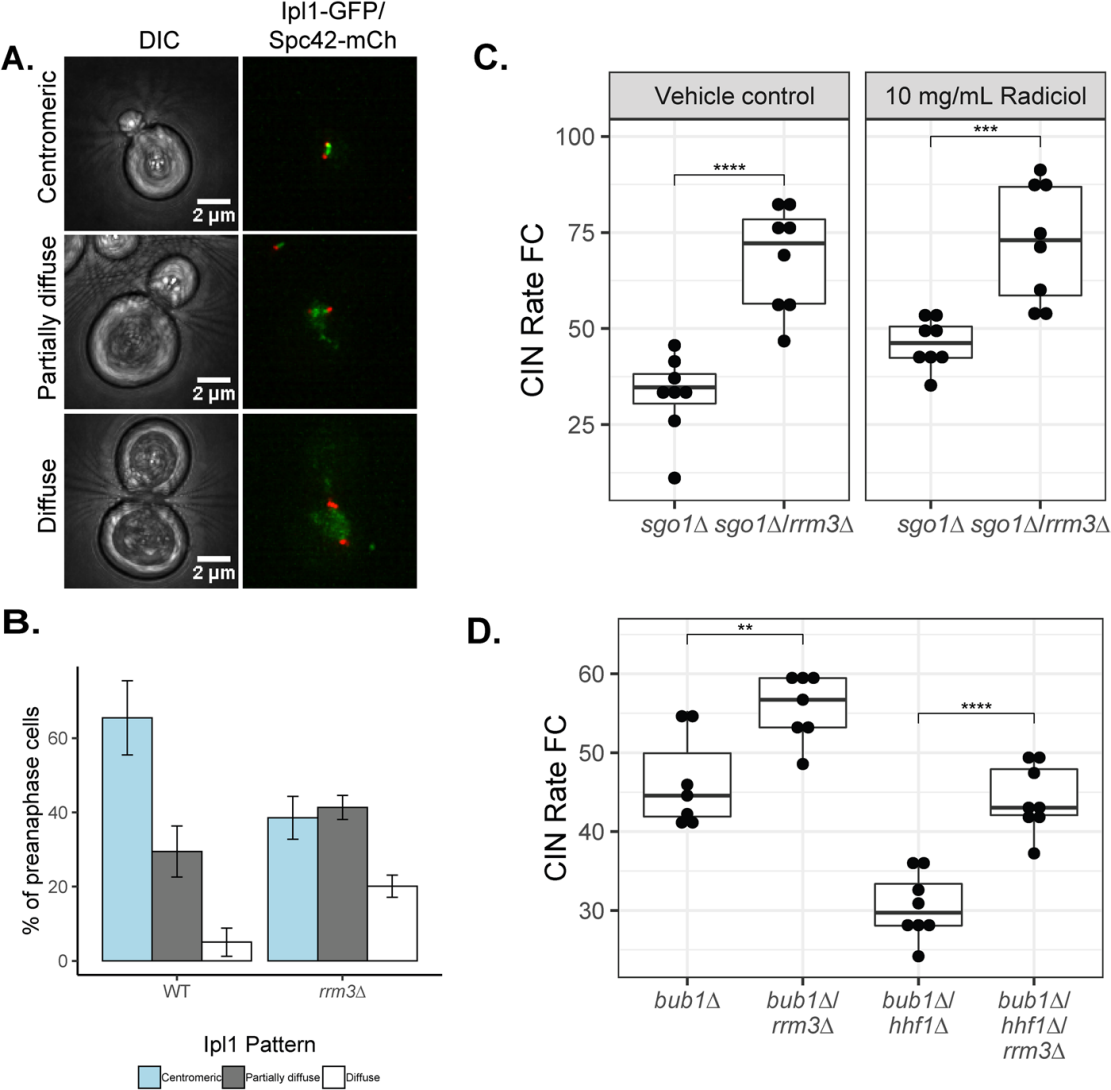
*rrm3∆* leads to perturbed distribution of Ipl1. (A) Representative images of different patterns of Ipl1-GFP localization to metaphase spindles. (B) Quantification of the percentage of cells with metaphase spindles has either centromeric, partially diffuse, or completely diffuse Ipl1-GFP signal in indicated strain. *n* = 64 and 122 cells for WT and *rrm3∆*, respectively, from three biological replicates. Error bars represent SEM (C) qCTF assay measuring CIN rates in *sgo1∆/rrm3∆* mutants in either DMSO (vehicle control) or 10 µg/ml Radicicol. (D) qCTF assay measuring the effect of *bub1∆* on the *rrm3∆*-dependent CIN suppression in an *hhf1∆* mutant. Box plots and statistical analysis are as in Figure 1.

Aside from the SAC and DDR, cells rely on robust error correction pathways to rectify improper kinetochore–microtubule attachments and ensure high-fidelity chromosome segregation. The chromosome passenger complex (CPC), which contains the Aurora B kinase (Ipl1 in yeast), is a crucial player in the error correction process by phosphorylating and subsequently promoting turnover of erroneous kinetochore-microtubule attachments (Vader *et al.*, 2006). To determine if Ipl1 function is altered in the *rrm3∆* mutant, we imaged Ipl1-GFP in an asynchronous population of WT or *rrm3∆* cells. Metaphase cells typically enrich Ipl1 between the poles of the bipolar spindle (Buvelot *et al.*, 2003). We found that the proportion of cells with exclusively centromere-localized Ipl1-GFP was reduced in *rrm3∆* cells to 38.5% compared with 65.5% in WT. Notably, while only 5.1% of WT cells exhibit a diffuse nuclear Ipl1-GFP signal in metaphase, this population was increased to 20.1% in *rrm3∆* cells (Figure 5, A and B).

Maintenance of Aurora B at the centromere depends on shugoshin (Sgo1 in yeast), and thus we next tested if Sgo1 is required for *rrm3∆*-dependent CIN suppression (Eshleman and Morgan, 2014; Peplowska *et al.*, 2014; Verzijlbergen *et al.*, 2014). *SGO1* deletion by itself elevated the CIN rate 29.7-fold compared with a WT strain, and the CIN rate was further increased in the *sgo1∆ rrm3∆* double mutant (Figure 5C). Because we were unable to generate viable a *sgo1∆ rrm3∆ hhf1∆* triple mutant, we tested if *sgo1∆* prevented *rrm3∆-*dependent CIN suppression in Radicicol-treated cells. Whereas *rrm3∆* suppresses CIN caused by Radicicol treatment (Figure 1C), this effect diminished when *SGO1* was deleted (Figure 5C). Sgo1 is localized to centromeric chromatin via Bub1 kinase activity upon phosphorylation of histone H2A at S121 (Fernius and Hardwick, 2007; Kawashima *et al.*, 2010). *rrm3∆* also elevated the CIN rate in the *bub1∆* background (Figure 5D). Furthermore, deletion of *bub1∆* prevented *rrm3∆*-dependent CIN suppression of the high-CIN mutant, *hhf1∆*, unlike the lack of effect by other SAC mutants tested (Figure 4). These results suggest that the Bub1-Sgo1 axis is important for CIN suppression by *rrm3∆*.

It was previously reported that *rrm3∆* cells are resistant to the microtubule poison benomyl (Chen *et al.*, 2019). Remarkably, we found that the *rrm3∆* mutant not only grows better than WT but can also rescue the lethality of *mad1∆* in the presence of benomyl, and this was not due to rescue of microtubule stability (Supplemental Figure S6, A and B). *rrm3∆* also leads to benomyl resistance in the high-CIN mutant *hhf1∆*. Growth in the presence of benomyl depends on either robust SAC signaling or microtubule–kinetochore attachment error correction pathways. These observations may be explained if *rrm3∆* enhances Ipl1-mediated error correction, and, if so, higher dosage of *IPL1* may phenocopy *rrm3∆* with respect to CIN suppression. To test this, we integrated an additional copy of the *IPL1* gene with endogenous promoter and terminator sequences into an ectopic locus on chromosome IV. We found that this additional copy of Aurora B kinase significantly suppressed CIN in the *hhf1∆* mutant (Supplemental Figure S6C). Of note, an additional copy of *IPL1* did not suppress CIN in the *csm3∆* mutant, which is not known to have any kinetochore–microtubule attachment errors (Supplemental Figure S6D). Overall, these results point toward improved error correction as one mechanism by which *rrm3∆* can lead to context-specific CIN suppression.

**FIGURE 6:**
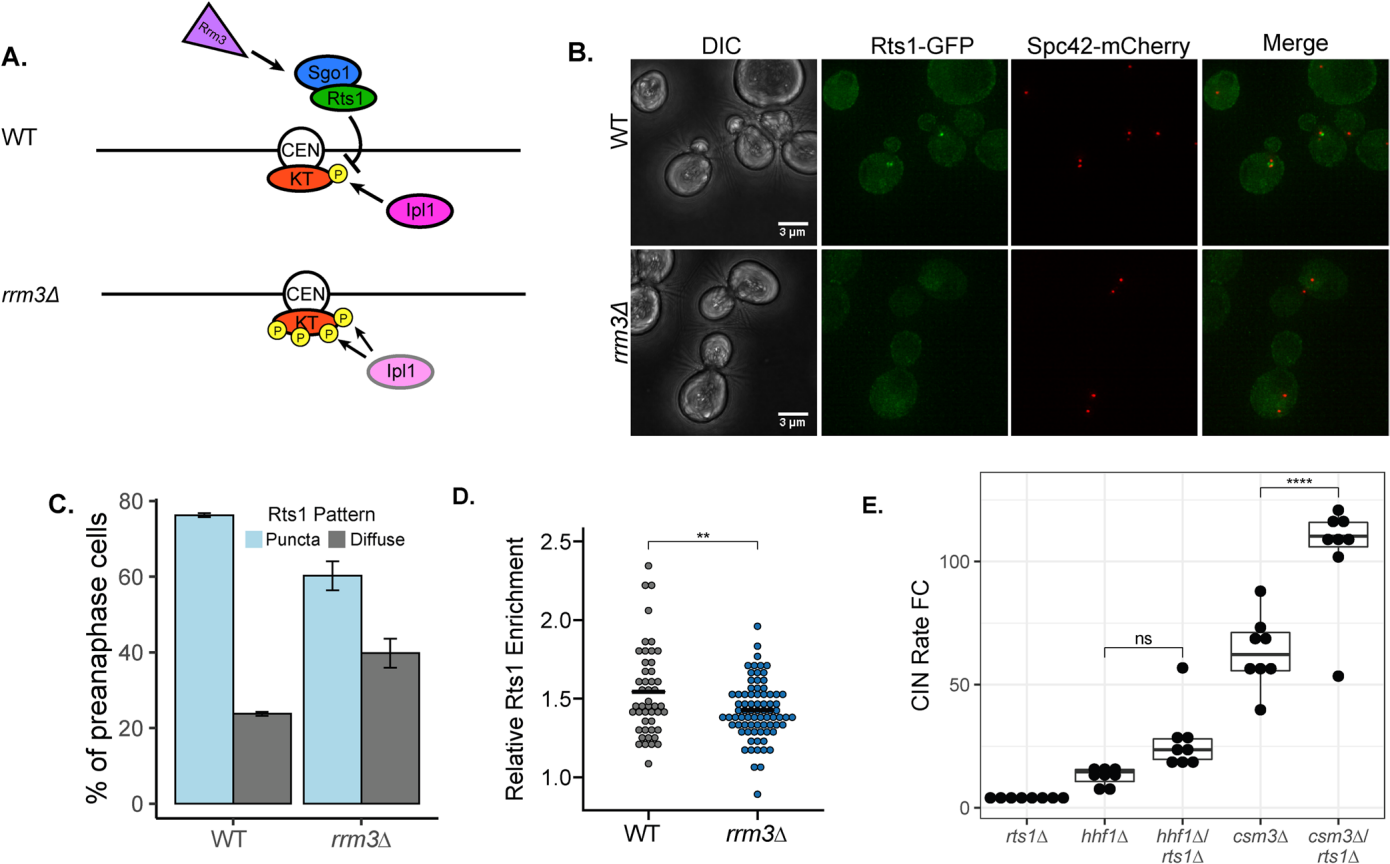
PP2A-Rts1 level at the centromere are perturbed in *rrm3∆* cells. (A) Cartoon model of impaired Sgo1-mediated Rts1 deposition onto centromeric DNA in the absence of Rrm3 leading to increased Ipl1 activity (yellow phosphorylation). For simplicity, only one chromatid is shown. Centromere: CEN, kinetochore: KT. (B) Representative images of Rts1-GFP localization to metaphase spindles in indicated strains. Quantification of (C) Rts1-GFP spindle enrichment in a box (width 500 nm) encompassing the entire spindle length relative to the total cellular level of Rts1-GFP. Black bar represents the mean of the data. (D) The percentage of metaphase cells containing either diffuse or puncta-localized Rts1-GFP signal at the spindle. *n* = 47 and 77 cells for WT and *rrm3∆*, respectively. Error bars represent SD of two biological replicates. (E) qCTF assay measuring the effect of *rts1∆* on CIN in the *csm3∆* or *hhf1∆* mutant. Box plots and statistical analysis are as in Figure 1.

How may altered Ipl1 localization contribute to suppressed CIN in the *rrm3∆* mutant? In addition to Ipl1, Sgo1 also recruits the regulatory subunit of protein phosphatase 2A (PP2A), Rts1, to the centromere to eventually dephosphorylate Aurora B kinase substrates (Eshleman and Morgan, 2014; Peplowska *et al.*, 2014). The presence of centromeric PP2A was shown to dampen the kinase activity of Aurora B in mammalian cells (Meppelink *et al.*, 2015). We therefore reasoned that Rts1 localization may also be altered in *rrm3∆* cells away from centromeres, which could lead to enhanced activity of Ipl1 despite a reduced centromeric pool of Ipl1 (Figure 6A). Under normal conditions, Rts1 has a cytoplasmic localization and becomes visibly concentrated at the centromere before anaphase onset (Gentry and Hallberg, 2002). In both WT and *rrm3∆* cells, Rts1 could be observed as puncta between the spindle poles, suggestive of centromeric localization, and diffuse throughout the cytoplasm. However, we found that the fraction of cells with Rts1 centromeric puncta was nearly twice as high in WT cells as in the *rrm3∆* mutant (Figure 6, B and C). In those *rrm3∆* mutant cells without Rts1 concentrated at the centromere, there was instead a diffusive GFP signal between the SPBs of preanaphase cells. Rts1-GFP enrichment between the SPBs of metaphase spindles was also significantly reduced in *rrm3∆* cells compared with that in WT (Figure 6D). However, complete loss of Rts1 (*rts1∆*) did not reduce the CIN rate of either *csm3∆* or *hhf1∆* mutants (Figure 6E).

## DISCUSSION

In this study, we performed a targeted qCTF screen to generate a ranked, quantitative list of the CIN rates of 324 yeast KO mutants (Table S3). Our data confirmed that several genes whose deletion caused high CIN rates (∼5-fold increases or more) encode proteins involved in processes not directly related to cell division, such as autophagy, endoplasmic reticulum inheritance, and mitochondria protein homeostasis. It will be interesting to investigate functional roles of these genes in chromosome segregation further. In our attempt to understand why *tof1∆* and *csm3∆* cause high CIN, we identified that deletion of the *RRM3* helicase leads to significant CIN suppression in these and a variety of other high CIN mutants (Table 1). This phenotype can be recapitulated by mutational ablation (*rrm3^K260A^*) of only Rrm3’s helicase activity. Interestingly, CIN suppression is specific to loss of function of Rrm3 but not that of the homologous helicase Pif1. At present it is unclear why deletion of only one helicase within the Pif1 family can lead to genome stability, but one reasonable possibility could be the nonoverlapping roles the helicases play at the centromere (Chen *et al.*, 2019).

Although both complete deletion of Rrm3 and *rrm3^K260A^* leads to a delayed S phase, S phase delay induced through HU treatment did not lead to CIN suppression. We further observed that neither the DDR nor SAC was required for *rrm3∆*-dependent CIN suppression in multiple CIN mutants tested. These findings appear different from the idea that, under certain contexts, an increase in cell cycle time allows improved error correction and genome stabilization (Vinton and Weinert, 2017). Perhaps when chromosome segregation errors are so pervasive, as with the high-CIN mutants tested in this current study, cells benefit more from increased activity of error-correcting mechanisms over increased time to correct the errors. In a related study, either mutating Rrm3 or treatment with HU in an otherwise WT background moderately elevates loss of a disomic chromosome VII, which is in line with evidence presented in this study (Admire *et al.*, 2006).

Indeed, we found specific differences with respect to the error correction pathway in the *rrm3∆* mutant. We first identified that this mutant harbors impaired maintenance of Aurora B kinase at centromeric DNA in metaphase cell populations. Similarly, the protein phosphatase PP2A-Rts1 that is responsible for dephosphorylating Ipl1 substrates also displayed altered centromere localization the Rrm3 mutant. These observations prompted us to explore shugoshin, which is required for centromere maintenance of both Aurora B kinase and PP2A-Rts1 (Peplowska *et al.*, 2014; Verzijlbergen *et al.*, 2014). Sgo1 itself was required for *rrm3∆*-dependent CIN suppression in Radicicol-treated cells. Supporting that the association of Sgo1 with chromatin is necessary for CIN suppression in *rrm3∆* mutants, Bub1, which localizes Sgo1 to chromatin, is also required for *rrm3∆*-dependent CIN suppression. This is not due to SAC disruption because other SAC mutants tested did not affect CIN suppression by *rrm3∆*.

Despite impaired Aurora B kinase localization, error correction in *rrm3∆* may be more effective than in WT. Increased error correction can result from PP2A depletion at the centromere in *rrm3∆*, which typically removes CPC-mediated phosphorylation through its phosphatase activity (Meppelink *et al.*, 2015). To this end, we found that the level of the yeast PP2A subunit, Rts1, at the centromere was reduced in the *rrm3∆* compared with WT. This interpretation is also consistent with the previous finding that in yeast centromeric association of Aurora B is not critical for error correction (Campbell and Desai, 2013). In line with the idea that error correction contributes to CIN suppression, we found that an extra copy of Aurora B kinase was sufficient to suppress CIN in the *hhf1∆* mutant. This is not the case in the *csm3∆* mutant, which has impaired sister chromatid cohesion establishment but no known link to kinetochore-microtubule attachment errors. This finding implies that *RRM3* deletion may suppress CIN through an alternative mechanism in the *CSM3* mutant, perhaps by allowing cells to pause replication at centromeres and properly load cohesin molecules.

In summary (Figure 6A), we propose that Rrm3 helicase activity somehow antagonizes the ability of shugoshin to accurately recruit or maintain substrates such as Ipl1 and Rts1 at centromeres. When Rrm3 helicase activity is inactivated, the PP2A-Rts1 subunit becomes titrated away from the centromere, leading to enhanced error correction via the pool of Aurora B kinase still present at the centromere. In addition to Sgo1, Aurora B kinase is targeted to centromeric chromatin by an Ndc10-mediated mechanism, which perhaps contributes to properly localized Ipl1 molecules even in the absence of the Rrm3 helicase (Yoon and Carbon, 1999; Cho and Harrison, 2011). A remaining question is if the chromatin localization patterns of Sgo1 and Rts1 change upon Rrm3 helicase inactivation, which may be revealed by using chromatin immunoprecipitation to quantify chromatin binding to non-centromeric regions in Rrm3 mutant strains.

Only a few previous studies identified methods to suppress CIN, such as increasing the cellular pool of nucleosides or cohesin, increasing the turnover of kinetochore–microtubule attachments by kinesin-13 overexpression, or delaying the rate of plus-end micro­tubule polymerization (Bakhoum *et al.*, 2008; Bester *et al.*, 2011; Burrell *et al.*, 2013; Manning *et al.*, 2014). In each case, the mechanism to suppress CIN was associated with a specific molecular mechanism that causes CIN. For example, mutation in the retinoblastoma protein led to a perturbed epigenetic signature that prevented the establishment of cohesion at the centromere, and an increased cohesin level suppressed CIN in this case (Manning *et al.*, 2014). Given that cancers can arise from various genetic underpinnings affecting different components required for faithful chromosome segregation, it is useful to identify pathways that can be manipulated to suppress CIN arising from many molecular perturbations. Our findings indicate that inactivation of Rrm3 helicase activity may be one method of suppressing CIN caused by diverse mutations that alter the proper centromere or kinetochore function. It will be interesting to investigate if inhibition of the Pif1 helicase in mammalian cells has a similar CIN suppression effect that may be exploited for cancer prevention or treatment.

## METHODS

Request a protocol through *Bio-protocol*.

### Yeast strains and growth conditions

Supplemental Tables S1 and S2 list yeast strains and plasmids used in this study, respectively. All KO, fluorescent, and epitope-tagged strains were constructed using PCR-based homologous recombination (Gietz and Schiestl, 2007). For Pif1 and Rrm3 mutants, the ORF was first replaced with a URA3 selectable marker, followed by replacement with the point mutant allele using an alternative selectable marker. All yeast strains were grown at 30°C unless otherwise noted.

qCTF strains were maintained in SD-Leu (Sunrise Biosciences, 1707-500) solid plates or media before the qCTF assay to select for the minichromosome. All other strains were grown in YPD unless otherwise noted. All drug treatments paired with qCTF assays were maintained for a 24-h culture period in SD-Complete medium (Sunrise Biosciences, 1701-500). qCTF assay drug concentrations were as follows: 10 µg/ml Radicicol (AG Scientific, R-1130), 25 mM Hydroxyurea (Sigma, H8627). For spot dilution assays, cells were grown to midlog phase in YPD to an OD of 0.62. A series of 10-fold dilutions was performed on this refreshed culture and 4 µl of each dilution was dropped onto indicated plates.

### Construction and validation of the “qCTF KO Library”

To generate the “qCTF KO Library,” we isolated genomic DNA from the Yeast Knock Out Library Mata collection using the 96-Well Plate Yeast Genomic DNA Mini-Preps Kit (BioBasic, BS8357). Following isolation, we performed high-throughput PCR to amplify the Kanamycin resistance cassette with 400 bp up- and downstream from the ORF using 5′/3′ untranslated region check primers designed using Primers-4-Yeast (Yofe and Schuldiner, 2014). PCR products were used in a transformation in combination with the Frozen-EZ Yeast Transformation II Kit (Zymo, T 2001) with 5 µl of PCR product added to the transformation mixture in a 96-well plate format. The transformation was carried out for 1 h at 30°C. Next, four volumes of SD-Leu media were added to the transformation mixture to allow an overnight outgrowth period with shaking at 250 rpm at 30°C. SD-Leu medium supplemented with 200 µg/ml G418 (Corning, 61-234-RG) was then directly added into the recovered liquid culture for a 1-wk selection. Because each of our mutants contained the Kan^R^ gene, we generated a control qCTF strain with Kan^R^ integrated in the TRP1 locus as a baseline to compare our mutants. The resulting strains were used to measure a “CIN rate,” which is defined as the frequency of MC loss/cell divisions. Our control qCTF strain had a CIN rate of ∼0.00016, equivalent to 1.6 MC missegregations per ten thousand divisions, which is comparable to our previous data and those of the standard CTF assay (Zhu *et al.*, 2015).

To assess the accuracy of mutant strain construction in our high-throughput qCTF-KO library, we genotyped a subset of 48 mutants that had over a twofold increase in CIN rate by isolating single colonies from the library and randomly selecting one colony to genotype by PCR. Each mutant was genotyped with an internal Kanamycin reverse primer paired with a primer ∼800 bp upstream from the gene of interest. We found 40/48 mutants to have correct genotypes through this validation, suggesting high efficiency of strain generation. Of the eight colonies that were not positively genotyped within the first round of PCR, five had a positive PCR genotyping product when we screened an additional three colonies/strain. As our high-throughput method does not contain a single colony isolation step, this validation suggests that most cells have the correct deletion, but there may be a small cohort of cells with off-target insertions of Kan^R^ construct. Based on this analysis, we did not filter out any samples from our screen results. In Supplemental Figure S1C, we present mutants with at least a fivefold increase in CIN rate. We separately validated the same cohort of 48 mutants genotyped by repeating the qCTF assay with seven or eight single random colonies extracted from our library (Supplemental Figure S1D). Supplemental Table S3 contains a complete list of the CIN rates of 328 qCTF-KO mutants and individually validated CIN rates. Overall, these secondary validation steps suggest that the data generated from our screen is a robust resource of quantified CIN rates of individual KO mutants.

### qCTF Assay

qCTF assay measurements and mathematical derivation of CIN rates were performed as described in Zhu *et al.* (2015) and Gordon *et al.* (2021). CIN rates measured for these mutants were converted to fold changes relative to a WT control. Unless otherwise noted, all box plots share these same parameters. Data points were only excluded if the number of GFP positive cells was counted to be lower than 10 or if the calculated CIN rate was negative.

### Fluorescent microscopy and quantification

Single colonies were inoculated into 3 ml of SC-complete medium (Sunrise Biosciences, 1459-100) and cultured at 250 rpm at 30°C overnight. The overnight cultures were diluted 1:100 in fresh SC-complete medium and grown for 3.5 h to a logarithmic phase. Between 1 and 2 ml of the refreshed culture was spun down at top speed in a tabletop centrifuge at room temperature for 1 min and concentrated into about 100 µl of residual medium. Concentrated log phase cells (1 µl) were applied to microscope slides for single–time point imaging. Images were acquired on a Spinning disk confocal microscope (60× oil immersion objective, 15 *Z*-slices covering a 7.5-µm range) or a GE DeltaVision OMX SR (60× oil immersion objective, 81 *Z*-slices covering a 10-µm range). For 3D time lapse microscopy, the concentrated cultures were applied to a 35-mm glass-bottomed dish (Mattek, P35G-0-14-C) treated with Concavalin A (Sigma, L7647) to immobilize the yeast. Movies were recorded on a spinning disc confocal microscope using a 2-min interval for a total of 180 min. All representative images are displayed as maximum intensity projections.

For cell cycle stage analysis in asynchronous cultures, we first stratified cells by bud status and classified all cells with no bud as G1. Cells with small buds and unduplicated SPBs (i.e., one Spc42-mCherry spot) were classified as S phase. For cells with two detectable Spc42-mCherry foci, Imaris spot detection software was used to quantify the distance between SPBs to differentiate between S phase and metaphase. Cells with interspindle >0 and <1.5 µm were classified as S phase and cells with interspindle distance >1.5 and <3 µm were classified as metaphase. All cells with spindles <3 µm were classified as anaphase.

For nuclear Pds1-GFP and Scc1-GFP quantification, maximum-intensity projected images of cells were used to identify cells manually with nuclear GFP signals. Nucleolar Cdc14 was defined as Cdc14-GFP signal overlapping with a nucleolar marker (Net1-mCherry) in undivided cells with metaphase spindles marked with Spc42-mCherry. For SPBD-to-anaphase onset analysis, Imaris spot detection software was used to identify the first time point at which a cell transitioned from one to two detectable SPBs. The time point of anaphase onset was defined as the spindle reaching a distance greater than 3 µm. As the *rrm3∆* mutant has a longer pre-anaphase spindle and sometimes crossed the 3-µm threshold before anaphase onset, we defined anaphase onset in this strain as the first time point at which the spindle exceeded 3 µm and increased in distance for three consecutive time points.

For identification of metaphase cells within asynchronous populations, we used an image analysis pipeline to distinguish automatically budded cells with two Spc42-mCherry foci separated by 0.5–3 µm. For Ipl1-GFP microscopy at the spindle, we manually defined the following categories: centromeric (GFP signal primarily detected between Spc42-mCherry bipolar spindle), partially diffuse (GFP signal was approximately equal at the spindle and nucleoplasm), and diffuse (GFP signal at spindle was indistinguishable from background nucleoplasmic signal). For relative enrichment of Rts1-GFP at the centromere, we drew a box of width 0.5 µm containing the entire length of the spindle on maximum-intensity projected images and measured the ratio of GFP intensity inside the box to the total cellular GFP, excluding the boxed region.

### DNA Content Analysis

Log phase yeast cultures were spun down at room temperature for 1 min at 13,200 rpm. Medium was removed and 1 ml of 70% ethanol was added dropwise while the pellet was shaken gently to fix cells. Samples were fixed overnight at 4°C. After fixation, 100 µl of fixed cells were spun down at 3000 rpm for 5 min at room temperature to remove ethanol, washed once with 0.5 M sodium citrate, and rehydrated for 10 min at room temperature. Samples were incubated overnight at 37°C in RNAse A solution (0.05 M sodium citrate with 0.1 mg/ml RNAse A). The sample was spun down at 3000 rpm for 5 min, and the supernatant was decanted. Samples were resuspended with Proteinase K solution (50 mM Tris HCl with 10 mM CaCl_2_ and 10 mg/ml proteinase K), and incubated at 55°C overnight. Samples were spun down and supernatants were removed, followed by resuspending cells in 0.5 ml of 0.05 M sodium citrate. Samples were sonicated for 3–4 s at the highest setting and stained with Sytox Green (Invitrogen, S7020) to a final concentration of 1 µM. Samples were analyzed on an Attune NxT Flow Cytometer 10 min after staining.

### Immunoblot analysis

Single colonies were inoculated into 3 ml of YPD media and cultured overnight at 30°C to generate “precultures.” Precultures were stored at room temperature and used for a maximum of 3 d. Precultures were diluted 1:10,000 in fresh YPD and shaken at 250 rpm at 30°C for 16 h to an OD_600_ of 0.15–0.3. Refreshed cultures were spun down at 4000 rpm for 5 min and washed two times with sterile room-temperature H_2_O. Cells were resuspended in 25 ml YPD supplemented with 25 µM α-factor (United Peptide). The culture was arrested for 3–4 h while rotating at 30°C. Once uniformly shmooing cells were confirmed, the samples were spun down and washed two times with ice-cold sterile water to remove the α-factor. The arrested cell population was released into 25 ml of fresh YPD and rotated at room temperature for 3 h. 1.5 ml of samples were spun down at labeled time points and washed in 750 µl ice-cold H_2_O followed by snap-freezing in liquid nitrogen. Samples were stored at –80°C before processing for immunoblot analysis.

Frozen cell pellets were processed by resuspension and boiling in 120 µl 1× LDS buffer (ThermoFischer Scientific, B0007) supplemented with 40 mM DTT for 10 min. Boiled cell lysates were subjected to bead beating with 0.5-mm glass beads (Sigma, Z250465) for 1.5 min at 4°C, followed by a final boiling for 10 min. Samples were pelleted for 1 min at top speed in a tabletop centrifuge at room temperature, and supernatant was separated from cell debris and beads. All lysates were run on a Bolt 4-12% Bis-Tris SDS–PAGE gel and transferred onto Bolt PDVF membranes using the iBlot 2 gel transfer device. Membranes were blocked for 1 h at room temperature with 5% BSA in 1× TBST and primary antibodies were incubated overnight at 4°C. The following primary antibodies were used to detect proteins of interest: HA (Cell Signaling Technologies, C29F4), Pgk1 (Invitrogen, 22C5D8).

### Budding index and DAPI-staining

Approximately 200 µl of cell cycle synchronized samples was collected and fixed for 15 min at room temperature in 100 µl of 4% paraformaldehyde made in 1× PBS. After fixation, samples were washed once with 500 KPO_4_/sorbitol buffer (final concentrations of 0.1 M potassium phosphate and 1.2 M sorbitol) and stored in 500 µl of KPO_4_/Sorbitol buffer at 4°C. Prior to imaging, samples were washed once with 1× PBS and stained with a final DAPI concentration of 1.25 µg/ml in 1× PBS. Arrested cells were imaged on a Nikon TiE-Eclipse epifluorescence microscope (60× oil immersion objective) and a single image capturing the middle of cells was used to assess the presence of a bud and nuclear division status. Cells with DAPI-stained nuclei clearly segregated to mother and daughter cells were categorized as anaphase cells. Between 16 and 145 cells were counted for each time point.

## Abbreviations used:

APC: anaphase-promoting complex
CIN: chromosome instability
DDR: DNA damage response
DRS: DNA replication stress
KO: knockout
qCTF: quantitative chromosome transmission fidelity
SAC: spindle assembly checkpoint.
