# Supplementary material for: Suppression of chromosome instability by targeting a DNA helicase in budding yeast
Source: Mol Biol Cell. 2022 Dec 15;34(1):ar3. doi: 10.1091/mbc.E22-09-0395 (PMC9816644; doi:10.1091/mbc.E22-09-0395)
Supplement: Supplementary file 1 [file mbc-34-ar3-s001.pdf]

Supplementary Materials

Molecular Biology of the Cell

Gordon *et al*.

Figure S1.

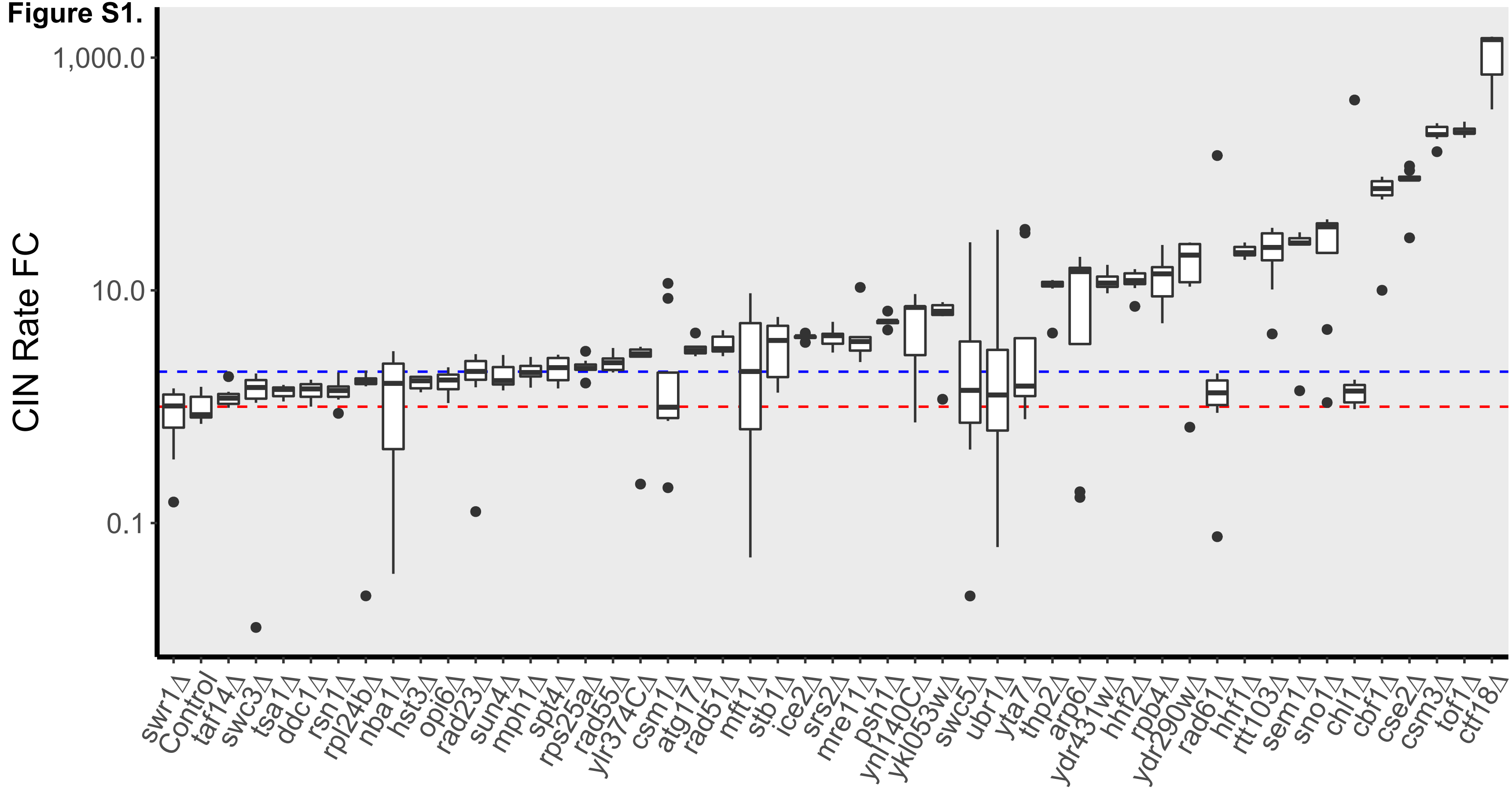
CIN Rate FC
1,000.0
10.0
0.1
swr1Δ
Control
taf14Δ
swc3Δ
tsa1Δ
ddc1Δ
rsn1Δ
rpl24bΔ
nba1Δ
hst3Δ
opi6Δ
rad23Δ
sun4Δ
mph1Δ
spt4Δ
rps25aΔ
rad55Δ
ylr374CΔ
csm1Δ
atg17Δ
rad51Δ
mft1Δ
stb1Δ
ice2Δ
srs2Δ
mre11Δ
psh1Δ
ynl140CΔ
ykl053wΔ
swc5Δ
ubr1Δ
yta7Δ
thp2Δ
arp6Δ
ydr431wΔ
hhf2Δ
rpb4Δ
ydr290wΔ
rad61Δ
hhf1Δ
rtt103Δ
sem1Δ
sno1Δ
chl1Δ
cbf1Δ
cse2Δ
csm3Δ
tof1Δ
ctf18Δ

Figure S2.

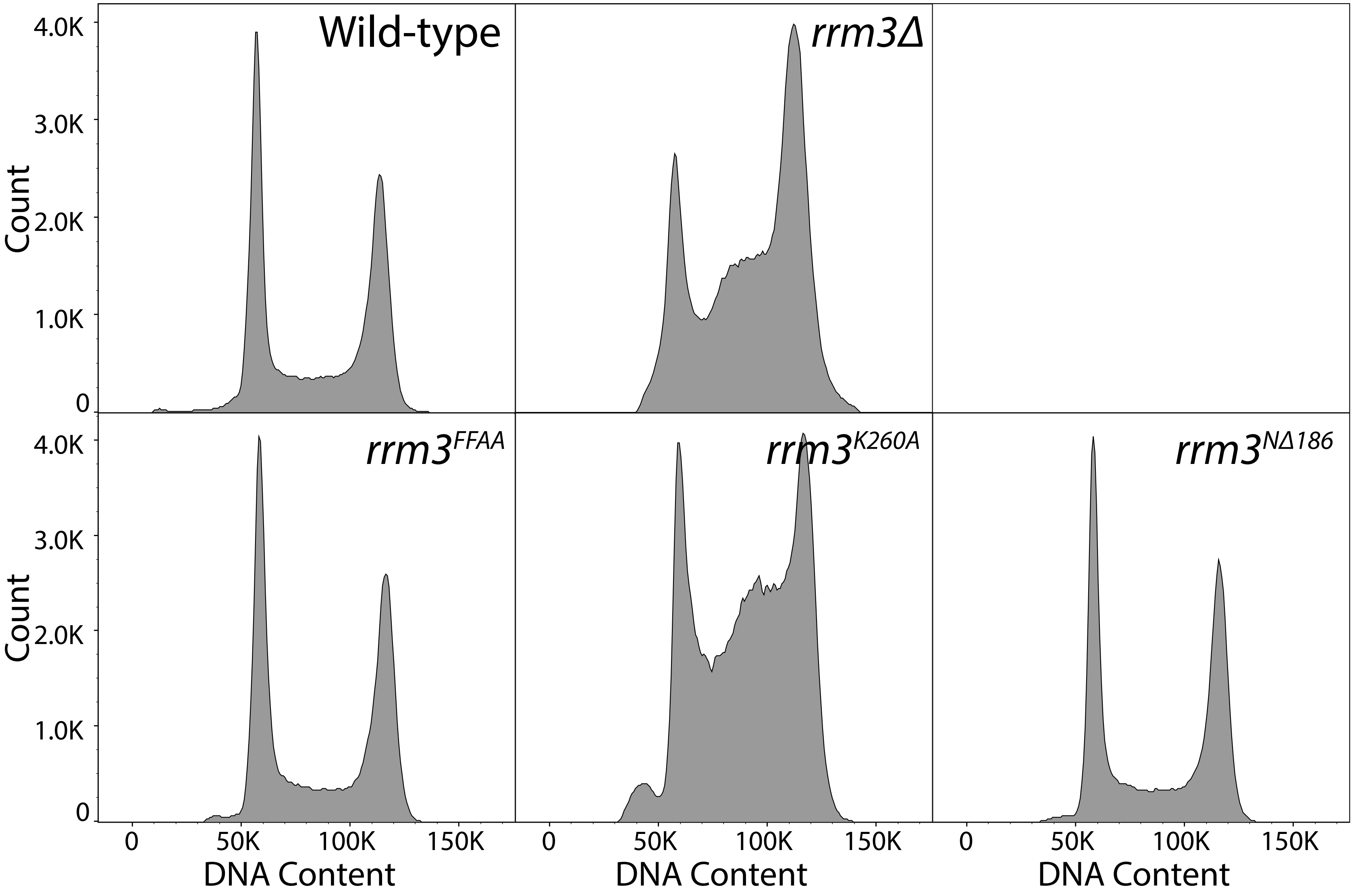

Wild-type
rrm3Δ
rrm3FFAA
rrm3K260A
rrm3NΔ186
Count
4.0K
3.0K
2.0K
1.0K
0
0
50K
100K
150K
DNA Content

Figure S3.

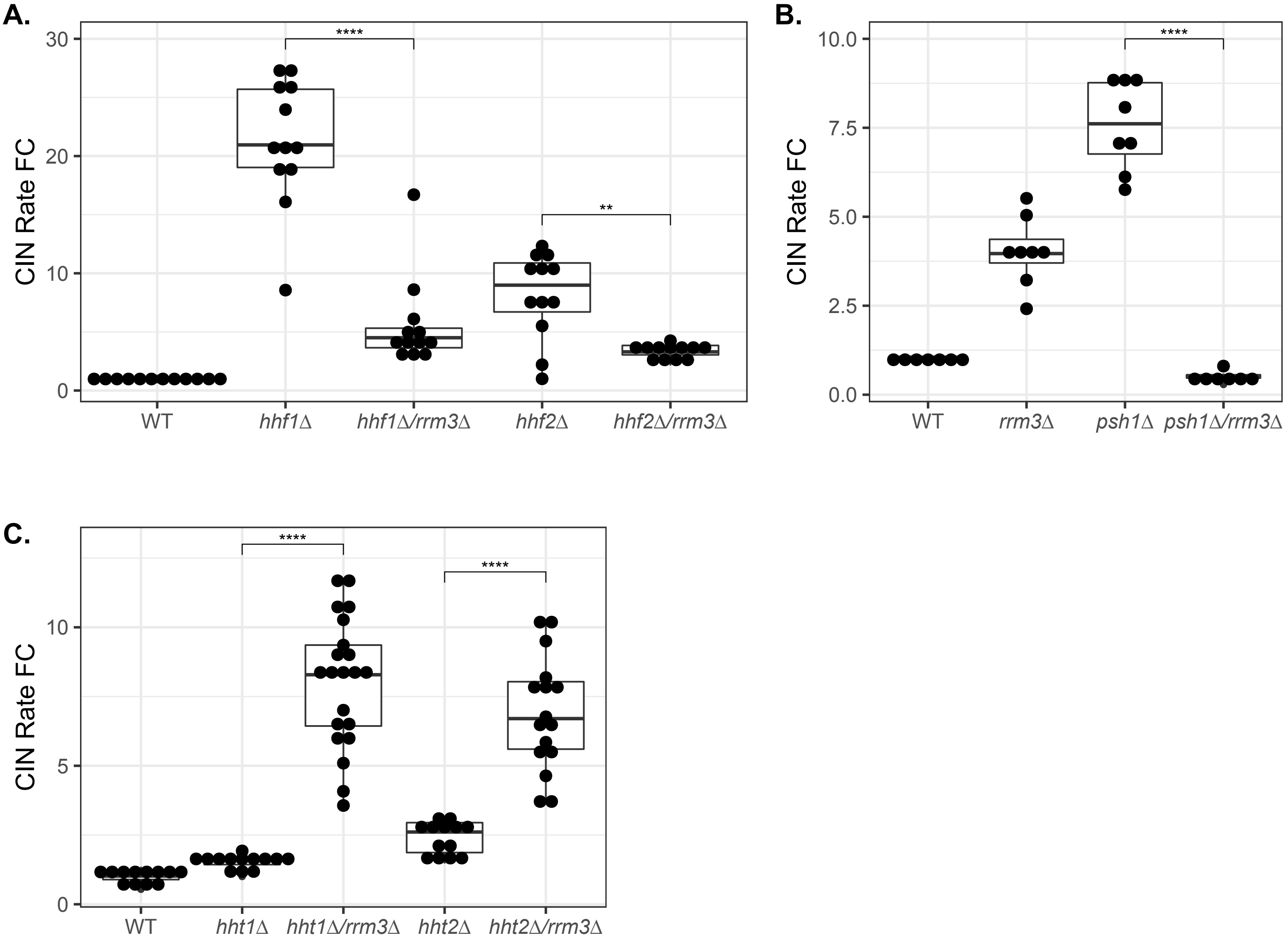

A.
CIN Rate FC
0
10
20
30
****
**
WT
hhf1Δ
hhf1Δ/rrm3Δ
hhf2Δ
hhf2Δ/rrm3Δ
B.
CIN Rate FC
0.0
2.5
5.0
7.5
10.0
****
WT
rrm3Δ
psh1Δ
psh1Δ/rrm3Δ
C.
CIN Rate FC
0
5
10
****
****
WT
hht1Δ
hht1Δ/rrm3Δ
hht2Δ
hht2Δ/rrm3Δ

Figure S4.

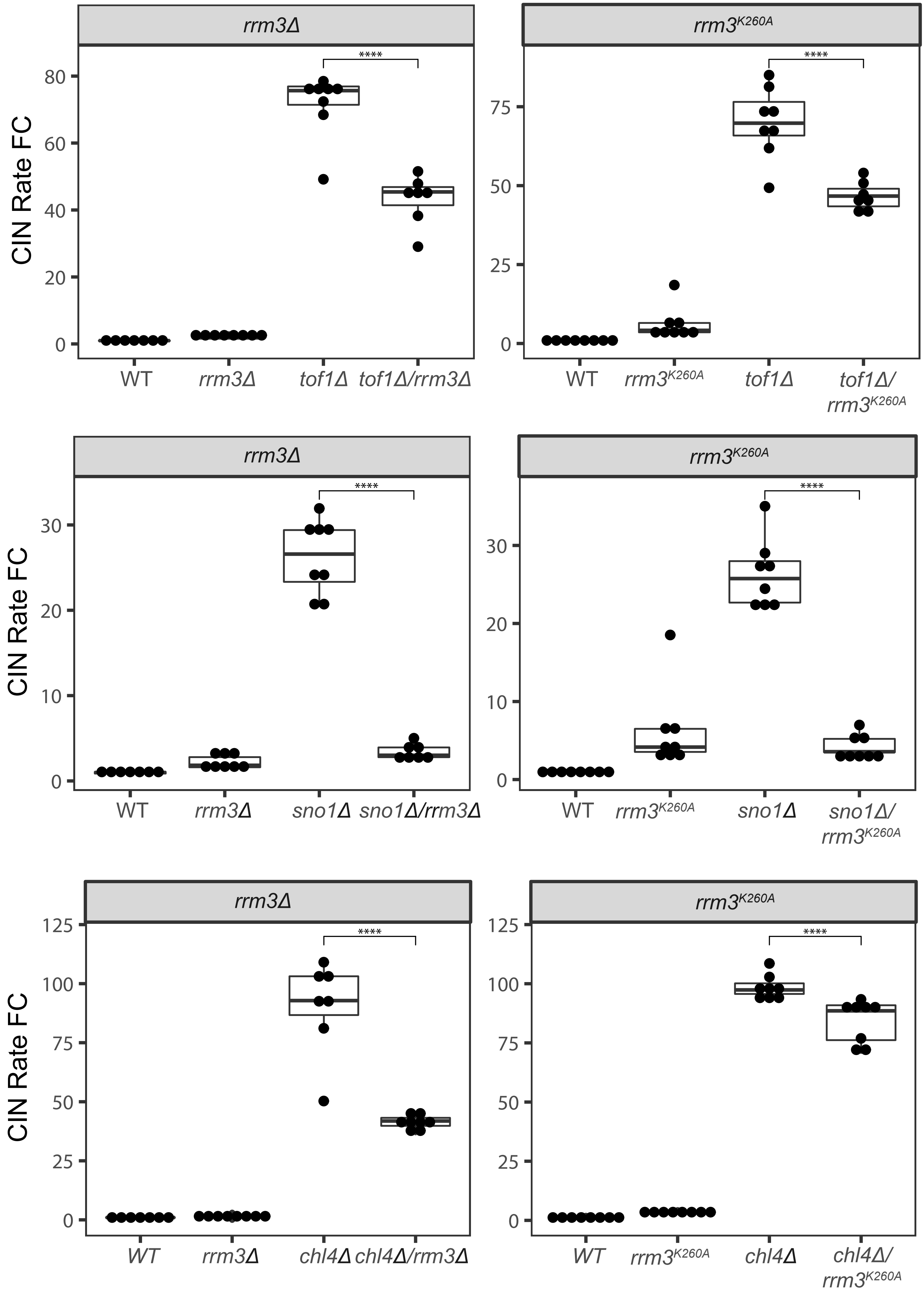
rrm3Δ
rrm3^K260A
CIN Rate FC
****
WT
rrm3Δ
tof1Δ
tof1Δ/rrm3Δ
rrm3^K260A
tof1Δ/ rrm3^K260A
sno1Δ
sno1Δ/rrm3Δ
sno1Δ/ rrm3^K260A
chl4Δ
chl4Δ/rrm3Δ
chl4Δ/ rrm3^K260A
0
20
40
60
80
25
50
75
10
30
100
125

Figure S5.

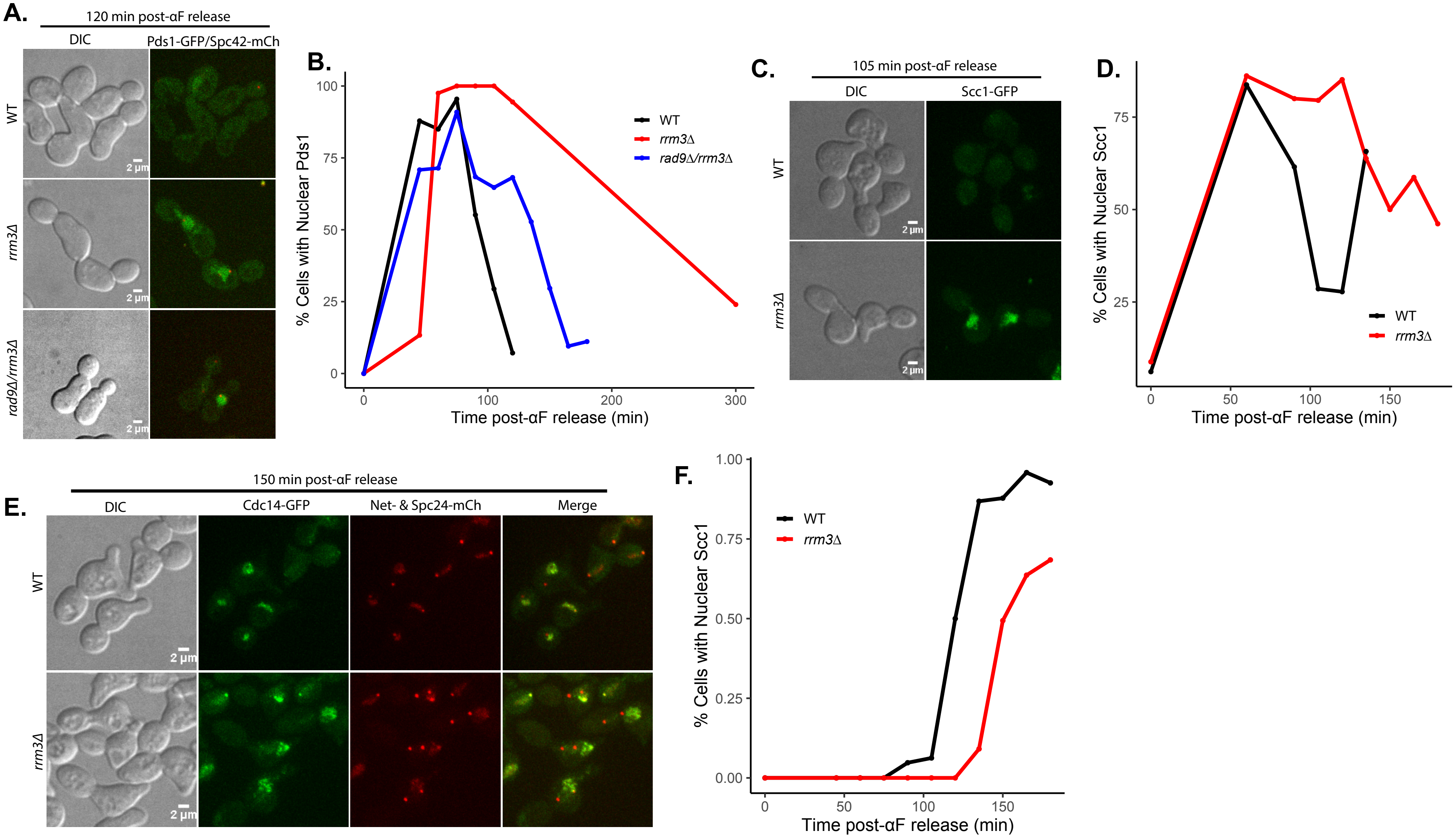
A.
120 min post-αF release
DIC
Pds1-GFP/Spc42-mCh
WT
rrm3Δ
rad9Δ/rrm3Δ
2 μm
B.
% Cells with Nuclear Pds1
Time post-αF release (min)
WT
rrm3Δ
rad9Δ/rrm3Δ
C.
105 min post-αF release
DIC
Scc1-GFP
WT
rrm3Δ
D.
% Cells with Nuclear Scc1
Time post-αF release (min)
E.
150 min post-αF release
DIC
Cdc14-GFP
Net- & Spc24-mCh
Merge
F.
% Cells with Nuclear Scc1
Time post-αF release (min)

Supplemental Figure 6.

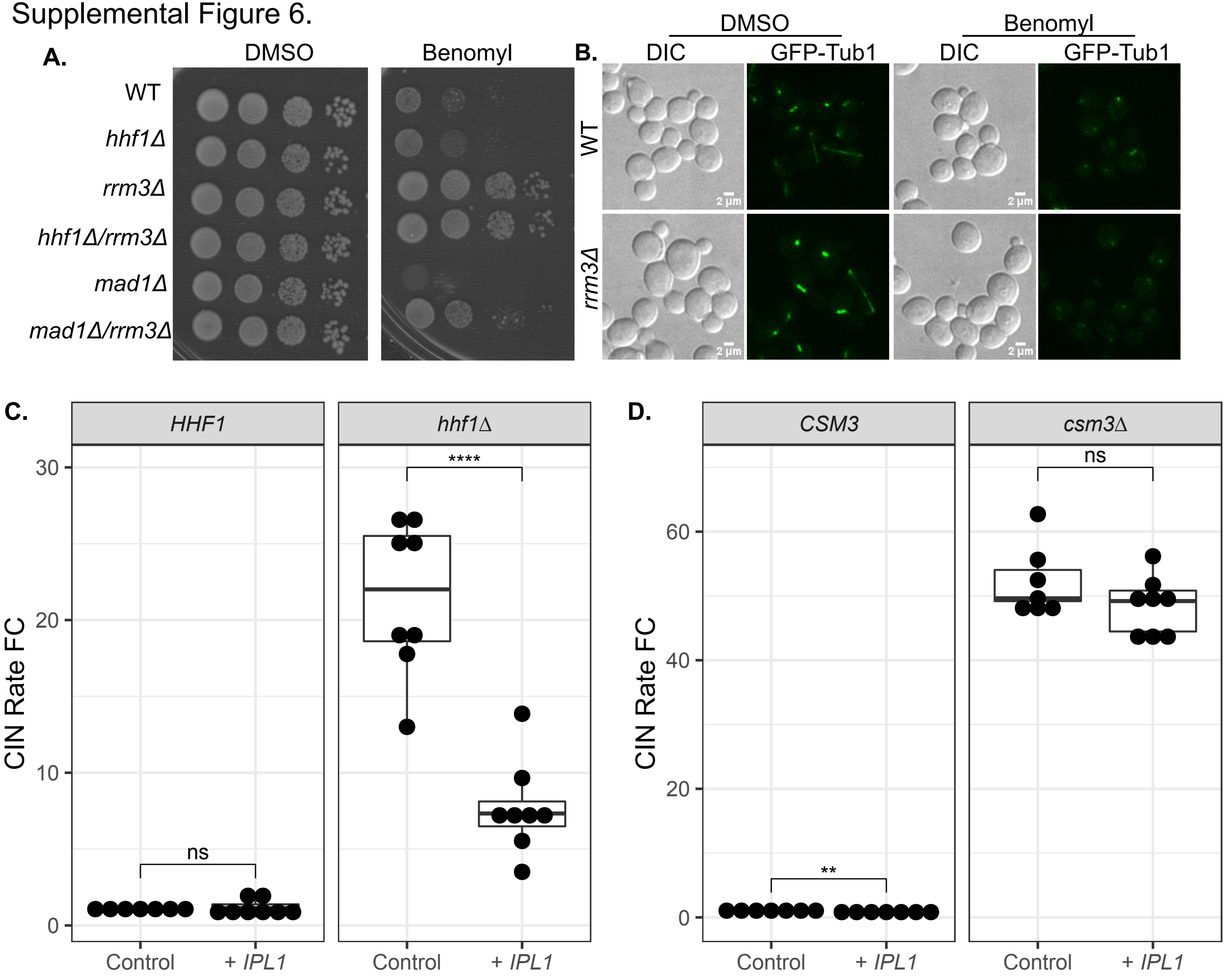

Fig. S1. Validation of the qCTF KO mutant screen. Between 3 to 8 biological replicates of the top 48 mutants from the qCTF-KO screen were used to calculate CIN rates in the qCTF assay. Data is displayed as a FC relative to a qCTF control strain. Data is arranged in increasing average FC. Red and blue dashed lines correspond to an FC of 1 and 2, respectively. Data beyond the end of the whiskers are plotted individually.

Fig. S2. Sytox green stained DNA content analysis on asynchronous cultures of wild type, *rrm3*Δ, *rrm3*^{FFAA}, *rrm3*^{K260A}, *rrm3*$^{N\Delta 186}$ backgrounds.

Fig. S3. Rrm3 deletion reduces CIN in histone H4 mutants but not histone H3 mutants. (A) qCTF measurement of the effect of rrm3Δ on the CIN rate of either Histone H4 mutant. **(B)** qCTF measurement of the effect of rrm3Δ on CIN rate in the psh1Δ mutant. **(C)** qCTF measurement of the effect of rrm3Δ on the CIN rate of either Histone H3 mutant. P-values for all plots were calculated from Tukey's posthoc test.

Fig. S4. Both the *rrm3*Δ and *rrm3*^{K260A} mutant suppresses CIN in multiple high CIN mutants. qCTF analysis measuring the effect of the *rrm3*^{K260A} mutation in *tof1*Δ **(A)**, *sno1*Δ **(B)**, and *chl4*Δ **(C)** backgrounds. Left plots show the effect of complete Rrm3 deletion on each high CIN mutant as control, while the right plots shows that of the *rrm3*^{K260A} mutation.

Fig. S5. Cell cycle differences in the *rrm3*Δ mutant. Nuclear Pds1-GFP **(A,B)**, nuclear Scc1-GFP **(C,D)**, and nucleolar Cdc14-GFP **(E,F)** were quantified in indicated strains at indicated time points post-αF release. The Cdc14-GFP also contained a Net1-mCherry and Spc42-mCherry marker to label the rDNA and spindle, respectively. Experiments were performed once with between 7 and 40 cells quantified per time point.

Fig. S6. *rrm3*Δ mutants are benomyl resistant. **(A)** Spot dilution spot assays of indicated mutants on YPD plates supplemented with either 30 µg/mL benomyl or an equivalent volume of DMSO. **(B)** Representative examples of WT and *rrm3*Δ mutant treated with DMSO or 30 µg/mL benomyl. Spindle is

marked with GFP-Tub1. Quantification of CIN rates of WT and *hhf1*Δ **(C)** or *csm3*Δ **(D)** mutants in the presence or absence of an additional integrated copy of *IPL1* ("+ IPL1"). Box plots and statistical analysis are as in Figure 1.
